# Functional Modulation of Human Macrophages by Secreted Phospholipases A_2_: Implications in Cancer

**DOI:** 10.3390/biomedicines10112763

**Published:** 2022-10-31

**Authors:** Maria Rosaria Galdiero, Ilaria Mormile, Francescopaolo Granata, Stefania Loffredo, Aikaterini Detoraki, Francesca Della Casa, Maria Luisa Trocchia, Annagioia Ventrici, Amato de Paulis, Francesca Wanda Rossi

**Affiliations:** 1 Department of Translational Medical Sciences, University of Naples Federico II, Via S. Pansini 5, 80131 Naples, Italy; 2 Center for Basic and Clinical Immunology Research (CISI), WAO Center of Excellence, University of Naples Federico II, Via S. Pansini 5, 80131 Naples, Italy; 3 Department of Internal Medicine, Clinical Immunology, Clinical Pathology, and Infectious Diseases, Azienda Ospedaliera Universitaria Federico II, Via S. Pansini 5, 80131 Naples, Italy; 4 Institute of Experimental Endocrinology and Oncology “G. Salvatore” (IEOS), National Research Council (CNR), Via S. Pansini 5, 80131 Naples, Italy

**Keywords:** cancer-related inflammation, tumor-associated macrophages, macrophage polarization, phospholipases

## Abstract

Cancer-related inflammation has recently emerged as an important component of cancer pathogenesis that is able to promote tumor initiation and progression, and the acquisition of the known hallmark capabilities, including evasion from immunosurveillance. Several soluble and cellular mediators participate in tumor microenvironment formation, leading to cancer initiation and progression. In this view, Tumor-Associated Macrophages (TAMs) are pivotal players and, due to their characteristic plasticity, can acquire a variety of distinct phenotypes and contribute in different ways to the different phases of carcinogenesis. Different stimuli have been shown to modulate macrophage polarization. Secreted phospholipase A_2_ enzymes (sPLA_2_s) exert multiple biological effects on cancer-related inflammation due to their enzymatic activity and ability to activate inflammatory cells by non-enzymatic mechanisms. Among the different sPLA_2_ isoforms, several studies have suggested that group IIA and group X are mainly involved in a wide variety of cancer types. A deeper insight into the molecular mechanisms regulating the link between tumor-infiltrating immune cells and cancer could lead to identifying new prognostic/predictive biomarkers and a broader view of cancer immunotherapy.

## 1. Introduction

In their seminal paper dated 2011, Hanahan and Weimberg revised their paradigm, shedding new light on the role of chronic inflammation as an enabling characteristic of cancer. Indeed, for a long time, the presence of an immune infiltrate within the tumor was attributed to the effort of the immune system to fight against tumors [1,2,3]. By contrast, in the last two decades, cancer-related inflammation (CRI) emerged as an important component of cancer, with the unexpected function of promoting tumor initiation and progression, supporting the acquisition of the known hallmark capabilities, including evasion from immunosurveillance. This evidence has dramatically changed the theoretical approach to cancer, transforming it from a “tumor cell centered” point of view to a point of view focused on the tumor microenvironment (TME) [2,3].

Among all components of the TME, Tumor-Associated Macrophages (TAMs) play a central role and are key orchestrators of CRI. Macrophage infiltration is evident in almost all tumor types, and these cells can represent the main mediators between inflammatory responses and cancer development and progression. Indeed, they are characterized by a peculiar plasticity, which consists in the ability to acquire distinct phenotypes and respond with specific functional outputs in response to signals derived from the microenvironment. In response to several stimuli, macrophages undergo reprogramming, which induces the development of a spectrum of distinct functional phenotypes [4,5]. Mimicking the Th1/Th2 paradigm, macrophages experience two different polarization states. Classically activated M1 macrophages are induced by interferon γ (IFNγ) alone or in concert with microbial stimuli (e.g., lipopolysaccharide (LPS)). In contrast, interleukin (IL)-4 and IL-13 inhibit this classical activation and induce the alternative M2 form of macrophage activation [6]. Classical M1 cells play a role as inducer and effector cells inTh1 responses and in mediating resistance against intracellular parasites and tumors. Indeed, they present an IL-12^high^, IL-23^high^, IL-10^low^ phenotype, and produce effectors molecules such as reactive oxygen and nitrogen intermediates and pro-inflammatory cytokines (e.g., IL-1β, tumor necrosis factor (TNF), IL-6) [5]. On the contrary, M2 macrophages display an IL-12^low^, IL-23^low^, IL-10^high^ phenotype and poor antigen-presenting capacity, suppress Th1 adaptive immunity, scavenge debris, take part in the dampening of inflammation, and promote wound healing, angiogenesis, tissue remodeling and tumor progression [4].

Phospholipase A_2_ (PLA_2_s) are mainly known as enzymes that hydrolyze the *sn*-2 acyl bond of glycerophospholipids (GPLs) to release lysophospholipids (LPLs) and free fatty acids (e.g., arachidonic acid), which are the main precursors of several biological active molecules, such as leukotrienes and prostaglandins [7,8,9]. Four major subfamilies of PLA_2_s are currently recognized: (1) high-molecular-weight cytosolic PLA_2_s (cPLA_2_s), (2) calcium-independent PLA_2_s (iPLA_2_s), (3) low-molecular-weight secreted PLA_2_s (sPLA_2_s), and (4) lipoprotein-associated PLA_2_ (Lp-PLA_2_). While cPLA_2_s, iPLA_2_s, and Lp-PLA_2_ mainly act as enzymes, sPLA_2_s can also act as extracellular mediators by exerting several additional functions, far beyond their enzymatic activity [10,11]. Currently, ten different isoforms of human sPLA_2_s have been identified. Some of them have been retrieved in TME, are widely reported to be involved in oncologic diseases and have a recognized role in human cancer [12,13].

In this review, we will recapitulate the main biological aspects of TAMs and their roles in cancer initiation and progression. We will explore the main functions of human sPLA_2_s in human cancers and the effects of human sPLA_2_s on macrophages in the context of cancer. Finally, we will discuss the roles of these multivalent mediators, and these tumor-infiltrating immune cells as means or targets of old and new anti-cancer therapeutic approaches.

## 2. Inflammation and Cancer: A Deadly Alliance

Inflammation is an ancestral physiological response aimed at containing damage and promoting tissue repair. In some conditions, this mechanism is circumvented, leading to chronic inflammation. In the case of a nascent neoplasia, the growing tumor can first elicit a physiological immune response, which, once bypassed, can give rise to a chronic inflammatory reaction, which prevents the resolution of the process and amplifies the cellular and molecular networks, sustaining the tumor growth and progression [1].

Chronic inflammation is now a well-recognized tumor-enabling capability, which can promote cancer development [2,14,15]. Tumors can generate an inflammatory response through several mechanisms. First, tumor cells can release chemotactic molecules that recruit innate immune cells, such as macrophages, mast cells and neutrophils [16]. In addition, the tumor can damage the normal tissue and induce the release of damage-associated molecular patterns (DAMPs), which further activate innate immune cells. These recruited cells release additional inflammatory molecules (e.g., sPLA_2_s), amplifying the response. Moreover, rising tumors increase the oxygen consumption due to their increasing metabolic request but, at the same time, can compromise blood and lymphatic vessels [17]. The resulting local hypoxia promote a metabolic switch, together with the production and the release of cytokines and angiogenic growth factors, which promote neo-angiogenesis and lymphangiogenesis and further recruit macrophages. These inflammatory networks persist as long as the tumor progress, thus, giving rise to a vicious cycle that is increasingly difficult to break.

## 3. Roles of TAMs in Tumor Growth and Progression

Within the TME, macrophages are the most represented leukocytes. In several human cancers such as breast, bladder and gastric cancer, high TAM infiltration was correlated with poor clinical outcome [18,19,20]. For instance, a high macrophage infiltration was associated with high tumor grade, lack of hormone receptor expression, and poor outcome in breast cancer patients [21]. Similarly, high TAM density correlated with advanced disease stage, vascular invasion, and poor survival in bladder cancer patients [22]. Quite recently, CD163+ TAM infiltration inversely correlated with overall survival in non-small cell lung cancer (NSCLC) patients undergoing immune checkpoint blockers treatment [23]. In hepatocellular carcinoma (HCC), peri-tumoral TAMs correlated with patient overall survival and disease-free survival [24]. In contrast, a positive correlation was observed between TAM infiltration and patient survival in high-grade osteosarcoma patients [25] and in gastric cancer TAMs positively correlated with tumor cell apoptosis and CD8^+^ infiltration [26]. Some apparently controversial results can be explained, considering that macrophage population within a tumor is not a homogeneous population, but TAM phenotype can vary within the same tumor. Moreover, there is huge variability on the technique used to identify TAMs in tissues (CD68^+^, CD203^+^, CD206^+^, stabilin1^+^ cells, etc.), which may account for the variability of the results among studies.

From a classical point of view, macrophages were considered terminally differentiated cells, deriving from circulating monocytes recruited at the tumor site, which undergo differentiation into macrophages under the influence of tumor-derived growth factors, such as macrophage colony-stimulating factor (M-CSF) or granulocyte-macrophage colony-stimulating factor (GM-CSF) [27]. However, this classic point of view has been revolutionized by the observation of a self-renewing population of macrophages, derived from embryonic precursors, that colonizes tissues before birth and is capable of locally proliferating and differentiating, independent from circulating monocytes [28,29,30,31,32,33,34]. To date, the relevance of macrophage proliferation in humans is still a matter of debate, and its contribution to cancer development is still unclear [21,35,36].

Tumor cells themselves as well as stromal cells can produce chemotactic molecules involved in monocyte recruitment at the tumor site, such as CC chemokine ligand 2 (CCL2) and CCL5, as well as growth factors such as vascular endothelial growth factor (VEGF) and M-CSF. Besides their chemotactic functions, these factors can also retain activating properties and contribute to macrophage polarization towards specific phenotypes within the TME [37]. In a murine model of transgenic mammary epithelial cell-specific CCL2 expressing mice, the overexpression of CCL2 in mammary tissue resulted in increased macrophage infiltration, increased expression of extracellular matrix (ECM)-remodeling genes (such as matrix metalloproteinases and lysyl oxidase) and increased stromal density and collagen deposition [38]. More recently, in a murine model of bladder cancer, radiotherapy induced the release of CCL2 by irradiated cancer cells, which in turn, promoted the recruitment of bone marrow-derived CCR2-positive myeloid cells and the polarization of M1-type TAMs toward the M2 type, suggesting a radio-resistance mechanism sustained by a CCL2-M2 macrophage network [39]. Moreover, the classical monocyte chemo-attractant and growth factor M-CSF favors macrophage survival and skewing towards a tumor-promoting “M2-like” phenotype [40,41].

Once in the TME, macrophage polarization can be driven by tumor cells, as well as by “tumor-educated” immune cells releasing M2-skewing factors such as IL-4, IL-13, immunocomplexes, transforming growth factor-β (TGF-β) or M-CSF. Interestingly, in TME, hypoxia leads to the extracellular accumulation of adenosine, which contributes to the generation of an immunosuppressive microenvironment, which in turn sustains cancer progression. One of the main immunosuppressive effects mediated by adenosine is the M2-skewing of TAMs through the engagement of A_2A_ receptors [42].

TAMs display a number of M2-like features in several tumor types, such as renal [43], breast [44], pancreatic [45], lung [46], and cervical cancer [47], and as described by in vivo and in vitro models [48,49,50]. TAMs are able to sustain tumor cell growth through the production of growth factors such as epidermal growth factor (EGF), which induce breast cancer cell proliferation [20]. TAMs generate high levels of reactive oxygen and nitrogen species, which contribute to DNA damage and the genetic instability of cancer cells [51]. Indeed, they release proteolytic enzymes, such as matrix metalloproteases, which are involved in ECM digestion and remodeling, thus, favoring tumor cell invasion and metastasis [52]. Moreover, TAMs are high producers of angiogenic/lymphangiogenic factors, such as VEGF-A, VEGF-C, TGF-β, as well as pro-angiogenic chemokines such as CCL2 and CXCL8 [53,54,55,56]. Interestingly, tumor-associated hypoxia, as well as through the high levels of adenosine that accumulate in the hypoxic microenvironment, can modulate TAMs phenotype and metabolic features, shifting the balance towards a pro-angiogenic and lymphangiogenic TME [56]. In an elegant in vivo model, under hypoxic conditions, TAMs upregulated FcγRIIb through the activation of HIF-1 and AP-1. These FcγRIIb^high^ TAMs displayed a reduced capability to eliminate anti-CD20 opsonized chronic lymphocytic leukemia cells in vitro, suggesting a role for hypoxia-driven TAM phenotype in immunotherapy resistance [57]. Finally, TAMs promote tumor progression by suppressing anti-tumor immunity. Indeed, TAMs produce immunosuppressive molecules such as TGF-β, IL-10, indoleamine 2,3-dioxygenase (IDO) and arginase-1, which suppress adaptive T-cell immune response and favor Treg recruitment and functions [58,59]. In a mouse model of Colitis-Associated Cancer (CAC), macrophages produced IL-17, which in turn, increased the survival and immunosuppressive activity of granulocytic myeloid-derived suppressor cells (MDSCs), thus, fostering tumor progression [60].

However, although TAMs resemble M2 macrophages in the vast majority of cancers, the TME represents the main example of the fine modulation of macrophage polarization states. Indeed, within the TME, TAMs acquire a wide range of activation states based on the cellular and molecular milieu, which characterizes different tumors and different tumor components (i.e., tumor cells, stromal cells and immune cells). Hence, the pathways of TAM activation vary among the various tumor types and, in some cases, within the same tumor [59,61]. For example, this is the case of the variable access to oxygen in the distinct tumor areas, which is responsible for various levels of activation of metabolic pathways (e.g., the adenosine pathway) involved in tuning macrophage phenotypes [44,62].

## 4. Roles of sPLAs in Cancer

The sPLA_2_s family includes ten different isoforms identified in human cells and tissues, which are referred to as group IB (hGIB), group IIA (hGIIA), group IID (hGIID), group IIE (hGIIE), group IIF (hGIIF), group III (hGIII), group V (hGV), group X (hGX), group XIIA (hGXIIA), and group XIIB (hGXIIB) [7]. sPLA_2_s are considered proinflammatory mediators since large amounts of these proteins have been observed in biological fluids during local or systemic inflammation [10]. In addition, sPLA_2_s are overexpressed in some acute or chronic inflammatory diseases of the lung (e.g., pneumonia, bronchial asthma, chronic obstructive pulmonary disease, and interstitial lung disease) [63], as well as in several types of cancer [13,64,65,66,67,68,69]. Although it has been reported that the expression of several sPLA_2_ isoforms (i.e., hGIB, hGIIA, hGIIE, hGIIF, hGV, hGX, hGXIIA) is increased in neoplastic tissues, two major sPLAs isoforms—namely, hGIIA and hGX—are particularly involved in a broad variety of cancer types [12,13,68,70,71].

hGIIA plays a dual role in cancer as it can both suppress and promote tumor progression, depending on the cancer type [13]. Indeed, it was positively associated with breast cancer recurrence, metastasis and death [72,73]. hGIIA exerts also a protumorigenic effect in lung cancer, acting through the nuclear factor of κB (NF-κB) activation [74] and in esophageal cancer. Indeed, the pharmacological or genetic inhibition of hGIIA in vitro decreased the proliferation of esophageal adenocarcinoma cells, while its overexpression resulted in enhanced cancer cell growth [75]. Conversely, GIIA plays an antitumorigenic role in gastric cancer, since its overexpression inhibited gastric cancer invasion and metastasis through the Wnt/β-catenin signaling pathway [76], and it has been associated with improved patient survival [70,77].

It was initially shown that hGX contributes to colon tumorigenesis by generating prostaglandin E2_2_ (PGE_2_) and other lipidic mediators [78,79]. In addition, mouse (m)GX is highly expressed in the mice colon, and it is the most potent enzyme to stimulate cell proliferation and the mitogen-activated protein kinase (MAPK) activation of various colon cancer cell lines [79]. This effect is mostly due to its intrinsic catalytic activity, which induces the production of free arachidonic acid and LPLs (which are mitogenic by themselves), large amounts of PGE2 and other eicosanoids from colon cancer cell lines. However, these lipid mediators do not play a role in mGX-induced cell proliferation because inhibitors of cyclooxygenases and lipoxygenases do not prevent sPLA_2_ mitogenic effects [79]. Quite recently, serum exosomal GX mRNA and protein have been associated with a more aggressive phenotype (i.e., higher stages, lymphatic node metastasis and distant metastasis) and poor overall and disease-free survival of NSCLC patients [80]. This sPLA_2_ has also been shown to play a role as a possible prognostic factor in other types of cancer. Indeed, recent findings by Kudo et al. demonstrate that the pro-tumorigenic action of lymphoma-derived extracellular vesicles (EVs), including exosomes, is augmented via sPLA_2_-driven lipid metabolism [81]. In particular, the hydrolysis of EV phospholipids by GX increased the production of fatty acids, LPLs, and their metabolites in the macrophages of Epstein-Barr virus lymphoma. The pharmacological inhibition of endogenous GX suppressed lymphoma growth in Epstein-Barr virus-infected humanized mice, whereas treatment with sPLA_2_-modified EVs reversed this phenotype [81]. Finally, hGX has been shown to facilitate the cell-cycle progression of soft tissue leiomyosarcoma. Indeed, a higher hGX expression significantly correlated with a worse relapse-free survival in soft tissue leiomyosarcoma patients [82].

A better understanding of the underlying mechanism behind the correlation between sPLA_2_s and cancer growth and progression will help to develop novel anticancer agents targeting sPLA_2_s [83,84]. The inhibition of sPLA_2_s can be considered as a novel advantageous strategy for preventing and treating inflammation-associated diseases and cancer [83,84,85].

## 5. Modulation of Macrophages Activated by Human PLA_2_s

sPLA_2_s exert multiple biological effects on CRI due to their enzymatic activity and ability to activate inflammatory cells by non-enzymatic mechanisms [10,86]. Macrophages are a major target of sPLA_2_s since they can be activated by both mechanisms [10,86]. Through their enzymatic activity, sPLA_2_s contribute to the biosynthesis of proinflammatory lipid mediators (PGs, leukotrienes, lipoxins, and platelet-activating factors) [7]. On the other hand, by non-enzymatic mechanisms involving the engagement of specific (M-type) or promiscuous receptors (mannose receptor and integrins), sPLA_2_s activate several functions including exocytosis, and the production of cytokines (TNF-α, IL-6, IL-10, and IL-12), chemokines (CCL-1, CCL2, CCL3, CCL4, and CXCL8) and angiogenic/lymphangiogenic factors (VEGF-A, VEGF-C), as well as the generation of nitrogen species and cell adhesion [56,87,88,89,90,91]. The receptor engagement leads to the activation of several intracellular pathways including the PI3K/Akt system, the MAPK p38, extracellular signal-regulated kinase 1/2 (ERK1/2), and NF-κB, which are involved in the production of cytokines, chemokines and angiogenic/lymphangiogenic factors [11,56,87,88]. Both human sPLA_2_s (i.e., hGIB, hGIIA, hGV, hGX) and snake venom sPLA_2_s (i.e., group IA and group III) isoforms are able to activate macrophages. Nevertheless, it is conceivable that in the TME, TAMs can be exposed to human sPLA_2_s produced by tumor cells, stromal cells or other tumor-infiltrating immune cells such as mast cells [12,13,92].

**Figure 1:**
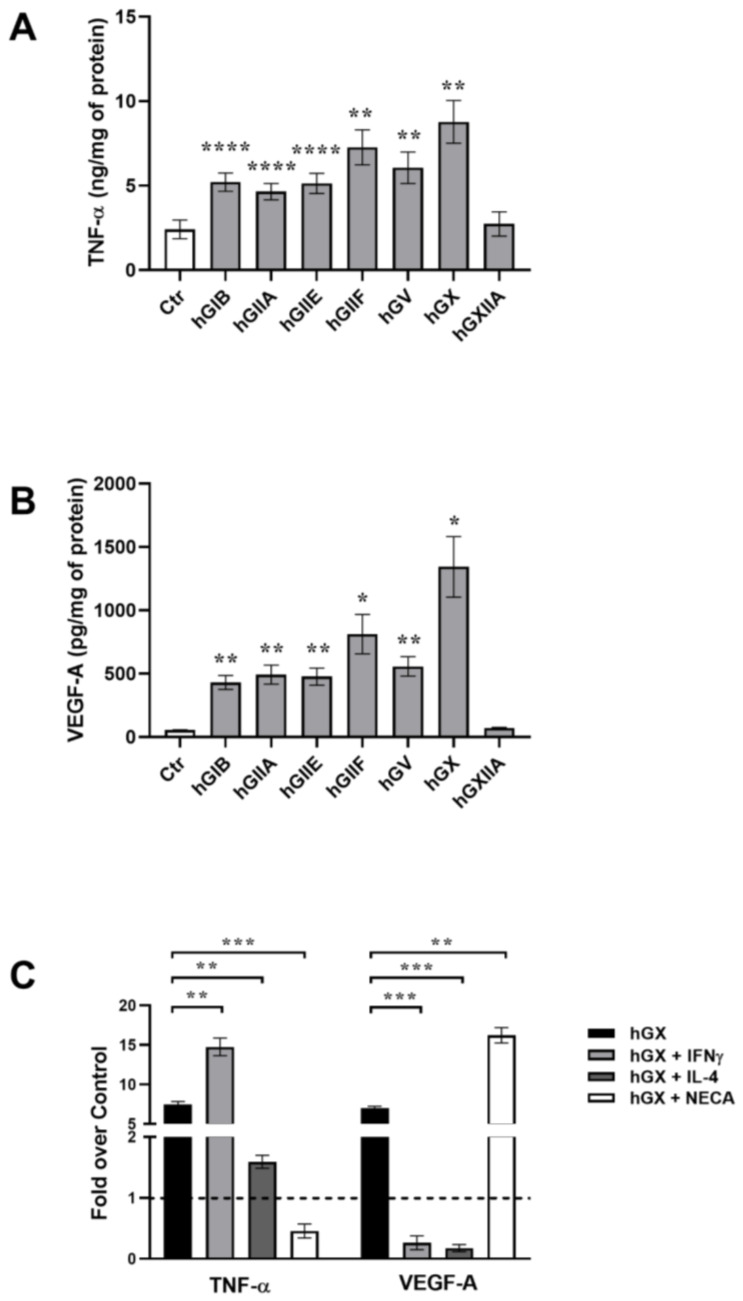
Different human-secreted phospholipase A2 enzymes (sPLA_2_) isoforms activate human lung macrophages (HLMs). HLMs were incubated (37 °C, 18 h) in RPMI 1640 containing human recombinant group IB (hGIB), group IIA (hGIIA), group IIE (hGIIE), group IIF (hGIIF), group V (hGV), group X (hGX), and group XIIA (hGXIIA) sPLA_2_s (kindly donated by G. Lambeau or M.H. Gelb). At the end of incubation, the supernatants were harvested, centrifuged (1000× *g*, 4 °C, 5 min) and stored at −80 °C for subsequent analyses. Tumor necrosis factor-α (TNF-α) (**A**) and vascular endothelial-derived growth factor-A (VEGF-A) (**B**) concentrations were determined by ELISA and values were normalized for the total protein (measured by Bradford assay) content in each well. The data are reported as the mean ± SEM of four different preparations of HLMs from four different donors. One-way ANOVA and Dunnett’s post test. * *p* < 0.05, ** *p* < 0.01, **** *p* < 0.001. vs. control. (**C**) Pro-inflammatory and angiogenic profiles in hGX-induced HLMs are differentially modulated by interferon-γ (INF-γ), interleukin (IL)-4, and 5’-(N-Ethylcarboxamido) adenosine (NECA). HLMs were incubated (37 °C, 18 h) in RPMI 1640 containing hGX (100 nM—black bars) alone or in combination with INF-γ (1000 U/mL—light gray bars), IL-4 (10 ng/mL—dark grey bars), or NECA (10 μM—white bars). At the end of incubation, the supernatants were harvested, centrifuged (1000× *g*, 4 °C, 5 min) and stored at −80 °C for subsequent analyses. TNF-α and VEGF-A concentrations were determined by ELISA and values were normalized for the total protein (measured by Bradford assay) content in each well. The data are reported as fold increase over control (unstimulated cells, dotted line), as the mean ± SEM of four different preparations of HLMs from four different donors. One-way ANOVA and Dunnett’s post test. * *p* < 0.05, ** *p* < 0.01, *** *p* < 0.005. vs. control.

To verify the last hypothesis, we performed some experiments to compare the ability of various human sPLA_2_s isoforms to activate and/or polarize human macrophages. We evaluated the effect of the equimolar concentrations (100 nM) of seven different human recombinant sPLA_2_s (hGIB, hGIIA, hGIIE, hGIIF, hGV, hGX, hGXIIA) on the production of TNF-α and VEGF-A from primary human lung macrophages (HLMs) (Granata et al., unpublished observation). Figure 1 shows that, with the exception of hGXIIA, all the sPLA_2_s induced a significant production of both TNF-α (A) and VEGF-A (B) from HLMs [91]. These results confirmed that most human sPLA_2_s were able to activate human macrophages to produce both M1-like, and M2-like soluble mediators.

Among the isoforms tested, hGX appears to be the most potent sPLA_2_ isoform able to activate HLMs. These results confirmed our previous observations suggesting that sPLA_2_s are pleiotropic mediators that induce a wide activation of macrophages but appear to be unable to induce the polarization towards a M1- or M2-like functional output (Granata et al., unpublished observation). The observation that hGXIIA was unable to induce TNF-α and VEGF-A from macrophages is not surprising because the functions of this isoform seem not to rely on enzymatic activity or on its binding properties [7,93,94].

Recently, we have shown that HLMs stimulated with a snake venom group, IA sPLA_2_ (svGIA), released a wide number of cytokines (TNF-α, IL-1β, IL-6, IL-10, IL-12,), chemokines (CXCL8, CCL1) and angiogenic factors (VEGF-A, ANGPT-1, ANG-PT-2) [91]. In line with previous reports, the M2-polarizing cytokine IL-4 inhibited the release of both TNF-α and VEGF-A induced by svGIA. Similarly, in line with its M1-polarizing effect [91], IFN-γ significantly enhanced TNF-α, whereas it abolished VEGF-A release from svGIA-activated macrophages. Finally, according to its M2-polarizing properties, the adenosine analog 5’-(N-Ethylcarboxamido) adenosine (NECA) significantly inhibited TNF-α, and greatly increased svGIA-induced VEGF-A release. However, in additional experiments, we observed that the M1- (IFN-γ) and M2-polarizing (IL-4) cytokines were unable to modify the release of TNF-α and VEGF-A from unstimulated (i.e., in the absence of sPLA_2_) HLMs (Granata et al., unpublished observation). These results indicate that macrophages can be preferentially modulated by M1/M2 polarizing stimuli when they are activated by a driving stimulus as sPLA_2_, adding a further piece to the complex puzzle of macrophage polarization states.

Since svGIA is of snake venom origin, it is unlikely that it can be retrieved at sites of tumor-growth inflammation together with M1/M2 polarizing stimuli. Therefore, we evaluated the effect of IFN-γ, IL-4 or adenosine on the release of TNF-α, and VEGF-A from HLMs activated by hGX. Figure 1C shows that IFN-γ increased hGX-induced the release of TNF-α but abolished the release of VEGF-A, according to its M1 polarizing properties. By contrast, the M2-polarizing molecule IL-4 strongly inhibited the release of both TNF-α and VEGF-A from hGX-activated macrophages, suggesting a wide inhibitory effect of this cytokine on macrophages stimulated with sPLA_2_. Finally, the adenosine analog NECA inhibited the hGX-induced release of TNF-α, but strongly increased VEGF-A release, according to its M2 polarizing properties. These observations confirm that human sPLA_2_s can play a role in the fine modulation of macrophage polarization and in the complex networks of the CRI.

**Figure 2:**
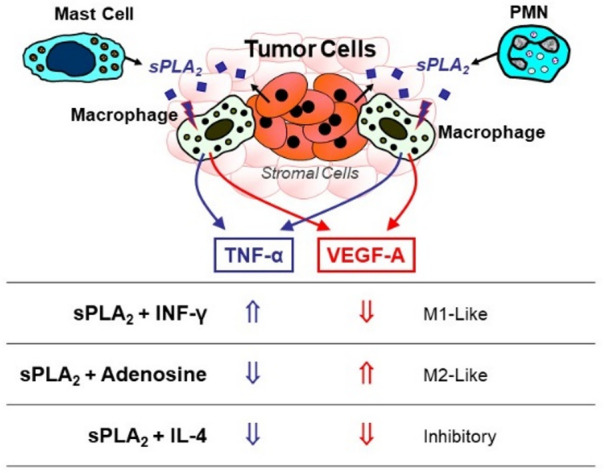
Hypothetical scheme depicting the complex dynamics of secreted phospholipase A2 enzymes (sPLA_2_s) and macrophage-polarizing stimuli within the tumor microenvironment (TME). Human sPLA_2_s released in the TME by tumor cells, stromal cells or other inflammatory cells infiltrating the tumor could act as a driving stimulus activating TAMs to produce cytokines, chemokines and angiogenic factors, without a clear polarization toward an M1- or M2-like functional output. In the presence of additional polarizing stimuli, such as interferon γ (IFNγ), interleukin (IL)-4 or adenosine, the phenotype of sPLA_2_-activated TAMs can be further fine-tuned, thereby modulating the functional output.

We can determine that human sPLA_2_s released in the TME by tumor cells, stromal cells or other inflammatory cells infiltrating the tumor (e.g., mast cells and neutrophils) could act as a driving stimulus activating TAMs to produce cytokines, chemokines and angiogenic factors, without a clear polarization toward an M1- or M2-like functional output (Figure 2). When in the TME, such polarizing stimuli as INF-γ, adenosine or IL-4 produced together with sPLA_2_s, can polarize the functional output of sPLA_2_-activated TAMs toward an M1-like (e.g., stimulation of TNF-α plus inhibition of VEGF-A), M2-like (e.g., inhibition of TNF-α plus stimulation of VEGF-A), or anti-inflammatory (e.g., inhibition of TNF-α and VEGF-A) phenotype to modulate tumor growth and progression.

## 6. Concluding Remarks

Increasing evidence shows that tumors grow and progress within their microenvironment, with which they constantly interact. Macrophages are integrated within CRI and can participate in the various phases of malignant initiation and progression. Cancer cells, and TAMs themselves, can release several pro-tumorigenic and pro-angiogenic cytokines and chemokines. These molecules could be a suitable target for inhibiting tumor growth by blocking pro-tumor functions. Indeed, enhancing anticancer immune responses by blocking the immunosuppressive molecules (i.e., IL-10, TGF-β, cytotoxic T-lymphocyte-associated antigen 4 (CTLA-4), programmed death 1 (PD-1), and PD-L1) expressed by cancer cells and tumor-infiltrating immune cells seems to be a promising therapeutic strategy in different types of cancers. Taken together, our results add a piece to the puzzle of the multiple roles for sPLA_2_s in the world of cancer-related macrophage polarization and pave the way for new sPLA_2_-targeting therapeutic tools [85]. In conclusion, a deeper insight into the molecular mechanisms regulating the link between sPLAs, macrophages, and cancer could lead to the identification of new prognostic/predictive biomarkers and a more personalized therapeutic approach.

## Data Availability

Not applicable.

## References

[1] Dvorak H.F. (1986). Tumors: Wounds that do not heal. Similarities between tumor stroma generation and wound healing. N. Engl. J. Med..

[2] Hanahan D., Weinberg R.A. (2011). Hallmarks of cancer: The next generation. Cell.

[3] Hanahan D., Weinberg R.A. (2000). The hallmarks of cancer. Cell.

[4] Biswas S.K., Mantovani A. (2010). Macrophage plasticity and interaction with lymphocyte subsets: Cancer as a paradigm. Nat. Immunol..

[5] Sica A., Mantovani A. (2012). Macrophage plasticity and polarization: In vivo veritas. J. Clin. Investig..

[6] Gordon S., Taylor P.R. (2005). Monocyte and macrophage heterogeneity. Nat. Rev. Immunol..

[7] Dennis E.A., Cao J., Hsu Y.H., Magrioti V., Kokotos G. (2011). Phospholipase A2 enzymes: Physical structure, biological function, disease implication, chemical inhibition, and therapeutic intervention. Chem. Rev..

[8] Murakami M., Sato H., Taketomi Y. (2020). Updating Phospholipase A2 Biology. Biomolecules.

[9] Vasquez A.M., Mouchlis V.D., Dennis E.A. (2018). Review of four major distinct types of human phospholipase A2. Adv. Biol. Regul..

[10] Triggiani M., Granata F., Giannattasio G., Marone G. (2005). Secretory phospholipases A2 in inflammatory and allergic diseases: Not just enzymes. J. Allergy Clin. Immunol..

[11] Ivanusec A., Sribar J., Krizaj I. (2022). Secreted Phospholipases A2—not just Enzymes: Revisited. Int. J. Biol. Sci..

[12] Scott K.F., Sajinovic M., Hein J., Nixdorf S., Galettis P., Liauw W., de Souza P., Dong Q., Graham G.G., Russell P.J. (2010). Emerging roles for phospholipase A2 enzymes in cancer. Biochimie.

[13] Peng Z., Chang Y., Fan J., Ji W., Su C. (2021). Phospholipase A2 superfamily in cancer. Cancer Lett..

[14] Balkwill F., Mantovani A. (2001). Inflammation and cancer: Back to Virchow?. Lancet.

[15] Balkwill F., Coussens L.M. (2004). Cancer: An inflammatory link. Nature.

[16] Bonavita E., Galdiero M.R., Jaillon S., Mantovani A. (2015). Phagocytes as Corrupted Policemen in Cancer-Related Inflammation. Adv. Cancer Res..

[17] Stylianopoulos T., Martin J.D., Chauhan V.P., Jain S.R., Diop-Frimpong B., Bardeesy N., Smith B.L., Ferrone C.R., Hornicek F.J., Boucher Y. (2012). Causes, consequences, and remedies for growth-induced solid stress in murine and human tumors. Proc. Natl. Acad. Sci. USA.

[18] Zhang Q.W., Liu L., Gong C.Y., Shi H.S., Zeng Y.H., Wang X.Z., Zhao Y.W., Wei Y.Q. (2012). Prognostic significance of tumor-associated macrophages in solid tumor: A meta-analysis of the literature. PLoS ONE.

[19] Bingle L., Brown N.J., Lewis C.E. (2002). The role of tumour-associated macrophages in tumour progression: Implications for new anticancer therapies. J. Pathol..

[20] Qian B.Z., Pollard J.W. (2010). Macrophage diversity enhances tumor progression and metastasis. Cell.

[21] Campbell M.J., Tonlaar N.Y., Garwood E.R., Huo D., Moore D.H., Khramtsov A.I., Au A., Baehner F., Chen Y., Malaka D.O. (2011). Proliferating macrophages associated with high grade, hormone receptor negative breast cancer and poor clinical outcome. Breast Cancer Res. Treat..

[22] Hanada T., Nakagawa M., Emoto A., Nomura T., Nasu N., Nomura Y. (2000). Prognostic value of tumor-associated macrophage count in human bladder cancer. Int. J. Urol. Off. J. Jpn. Urol. Assoc..

[23] Larroquette M., Guegan J.P., Besse B., Cousin S., Brunet M., Le Moulec S., Le Loarer F., Rey C., Soria J.C., Barlesi F. (2022). Spatial transcriptomics of macrophage infiltration in non-small cell lung cancer reveals determinants of sensitivity and resistance to anti-PD1/PD-L1 antibodies. J. Immunother. Cancer..

[24] Yusa T., Yamashita Y.I., Okabe H., Nakao Y., Itoyama R., Kitano Y., Kaida T., Miyata T., Mima K., Imai K. (2022). Survival impact of immune cells infiltrating peri-tumoral area of hepatocellular carcinoma. Cancer Sci..

[25] Buddingh E.P., Kuijjer M.L., Duim R.A., Burger H., Agelopoulos K., Myklebost O., Serra M., Mertens F., Hogendoorn P.C., Lankester A.C. (2011). Tumor-infiltrating macrophages are associated with metastasis suppression in high-grade osteosarcoma: A rationale for treatment with macrophage activating agents. Clin. Cancer. Res..

[26] Ohno S., Inagawa H., Dhar D.K., Fujii T., Ueda S., Tachibana M., Suzuki N., Inoue M., Soma G., Nagasue N. (2003). The degree of macrophage infiltration into the cancer cell nest is a significant predictor of survival in gastric cancer patients. Anticancer. Res..

[27] Allavena P., Sica A., Garlanda C., Mantovani A. (2008). The Yin-Yang of tumor-associated macrophages in neoplastic progression and immune surveillance. Immunol. Rev..

[28] Jenkins S.J., Ruckerl D., Cook P.C., Jones L.H., Finkelman F.D., van Rooijen N., MacDonald A.S., Allen J.E. (2011). Local macrophage proliferation, rather than recruitment from the blood, is a signature of TH2 inflammation. Science.

[29] Davies L.C., Rosas M., Smith P.J., Fraser D.J., Jones S.A., Taylor P.R. (2011). A quantifiable proliferative burst of tissue macrophages restores homeostatic macrophage populations after acute inflammation. Eur. J. Immunol..

[30] Robbins C.S., Hilgendorf I., Weber G.F., Theurl I., Iwamoto Y., Figueiredo J.L., Gorbatov R., Sukhova G.K., Gerhardt L.M., Smyth D. (2013). Local proliferation dominates lesional macrophage accumulation in atherosclerosis. Nat. Med..

[31] Ginhoux F., Schultze J.L., Murray P.J., Ochando J., Biswas S.K. (2016). New insights into the multidimensional concept of macrophage ontogeny, activation and function. Nat. Immunol..

[32] Weinberger T., Esfandyari D., Messerer D., Percin G., Schleifer C., Thaler R., Liu L., Stremmel C., Schneider V., Vagnozzi R.J. (2020). Ontogeny of arterial macrophages defines their functions in homeostasis and inflammation. Nat. Commun..

[33] De Couto G. (2019). Macrophages in cardiac repair: Environmental cues and therapeutic strategies. Exp. Mol. Med..

[34] Augusto-Oliveira M., Arrifano G.P., Lopes-Araujo A., Santos-Sacramento L., Takeda P.Y., Anthony D.C., Malva J.O., Crespo-Lopez M.E. (2019). What Do Microglia Really Do in Healthy Adult Brain?. Cells.

[35] Lutgens E., de Muinck E.D., Kitslaar P.J., Tordoir J.H., Wellens H.J., Daemen M.J. (1999). Biphasic pattern of cell turnover characterizes the progression from fatty streaks to ruptured human atherosclerotic plaques. Cardiovasc. Res..

[36] Bottazzi B., Erba E., Nobili N., Fazioli F., Rambaldi A., Mantovani A. (1990). A paracrine circuit in the regulation of the proliferation of macrophages infiltrating murine sarcomas. J. Immunol..

[37] Kitamura T., Qian B.Z., Soong D., Cassetta L., Noy R., Sugano G., Kato Y., Li J., Pollard J.W. (2015). CCL2-induced chemokine cascade promotes breast cancer metastasis by enhancing retention of metastasis-associated macrophages. J. Exp. Med..

[38] Sun X., Glynn D.J., Hodson L.J., Huo C., Britt K., Thompson E.W., Woolford L., Evdokiou A., Pollard J.W., Robertson S.A. (2017). CCL2-driven inflammation increases mammary gland stromal density and cancer susceptibility in a transgenic mouse model. Breast Cancer Res. BCR.

[39] Chiang Y., Tsai Y.C., Wang C.C., Hsueh F.J., Huang C.Y., Chung S.D., Chen C.H., Pu Y.S., Cheng J.C. (2022). Tumor-derived C-C motif ligand 2 (CCL2) induces the recruitment and polarization of tumor-associated macrophages and increases the metastatic potential of bladder cancer cells in the postirradiated microenvironment. Int. J. Radiat. Oncol. Biol. Phys..

[40] Pyonteck S.M., Akkari L., Schuhmacher A.J., Bowman R.L., Sevenich L., Quail D.F., Olson O.C., Quick M.L., Huse J.T., Teijeiro V. (2013). CSF-1R inhibition alters macrophage polarization and blocks glioma progression. Nat. Med..

[41] Van Overmeire E., Stijlemans B., Heymann F., Keirsse J., Morias Y., Elkrim Y., Brys L., Abels C., Lahmar Q., Ergen C. (2016). M-CSF and GM-CSF Receptor Signaling Differentially Regulate Monocyte Maturation and Macrophage Polarization in the Tumor Microenvironment. Cancer Res..

[42] Antonioli L., Fornai M., Pellegrini C., D’Antongiovanni V., Turiello R., Morello S., Hasko G., Blandizzi C. (2021). Adenosine Signaling in the Tumor Microenvironment. Adv. Exp. Med. Biol..

[43] Daurkin I., Eruslanov E., Stoffs T., Perrin G.Q., Algood C., Gilbert S.M., Rosser C.J., Su L.M., Vieweg J., Kusmartsev S. (2011). Tumor-associated macrophages mediate immunosuppression in the renal cancer microenvironment by activating the 15-lipoxygenase-2 pathway. Cancer Res..

[44] Movahedi K., Laoui D., Gysemans C., Baeten M., Stange G., Van den Bossche J., Mack M., Pipeleers D., In’t Veld P., De Baetselier P. (2010). Different tumor microenvironments contain functionally distinct subsets of macrophages derived from Ly6C(high) monocytes. Cancer Res..

[45] Gocheva V., Wang H.W., Gadea B.B., Shree T., Hunter K.E., Garfall A.L., Berman T., Joyce J.A. (2010). IL-4 induces cathepsin protease activity in tumor-associated macrophages to promote cancer growth and invasion. Genes Dev..

[46] Koukourakis M.I., Giatromanolaki A., Kakolyris S., O’Byrne K.J., Apostolikas N., Skarlatos J., Gatter K.C., Harris A.L. (1998). Different patterns of stromal and cancer cell thymidine phosphorylase reactivity in non-small-cell lung cancer: Impact on tumour neoangiogenesis and survival. Br. J. Cancer.

[47] Schoppmann S.F., Birner P., Stockl J., Kalt R., Ullrich R., Caucig C., Kriehuber E., Nagy K., Alitalo K., Kerjaschki D. (2002). Tumor-associated macrophages express lymphatic endothelial growth factors and are related to peritumoral lymphangiogenesis. Am. J. Pathol..

[48] Vasiljeva O., Papazoglou A., Kruger A., Brodoefel H., Korovin M., Deussing J., Augustin N., Nielsen B.S., Almholt K., Bogyo M. (2006). Tumor cell-derived and macrophage-derived cathepsin B promotes progression and lung metastasis of mammary cancer. Cancer Res..

[49] DeNardo D.G., Barreto J.B., Andreu P., Vasquez L., Tawfik D., Kolhatkar N., Coussens L.M. (2009). CD4(+) T cells regulate pulmonary metastasis of mammary carcinomas by enhancing protumor properties of macrophages. Cancer Cell.

[50] Joyce J.A., Pollard J.W. (2009). Microenvironmental regulation of metastasis. Nat. Rev. Cancer.

[51] Bonavita E., Gentile S., Rubino M., Maina V., Papait R., Kunderfranco P., Greco C., Feruglio F., Molgora M., Laface I. (2015). PTX3 is an extrinsic oncosuppressor regulating complement-dependent inflammation in cancer. Cell.

[52] Allavena P., Mantovani A. (2012). Immunology in the clinic review series; focus on cancer: Tumour-associated macrophages: Undisputed stars of the inflammatory tumour microenvironment. Clin. Exp. Immunol..

[53] Hotchkiss K.A., Ashton A.W., Klein R.S., Lenzi M.L., Zhu G.H., Schwartz E.L. (2003). Mechanisms by which tumor cells and monocytes expressing the angiogenic factor thymidine phosphorylase mediate human endothelial cell migration. Cancer Res..

[54] Schmidt T., Carmeliet P. (2010). Blood-vessel formation: Bridges that guide and unite. Nature.

[55] Murdoch C., Muthana M., Coffelt S.B., Lewis C.E. (2008). The role of myeloid cells in the promotion of tumour angiogenesis. Nat. Rev. Cancer.

[56] Granata F., Frattini A., Loffredo S., Staiano R.I., Petraroli A., Ribatti D., Oslund R., Gelb M.H., Lambeau G., Marone G. (2010). Production of vascular endothelial growth factors from human lung macrophages induced by group IIA and group X secreted phospholipases A2. J. Immunol..

[57] Hussain K., Liu R., Smith R.C.G., Muller K.T.J., Ghorbani M., Macari S., Cleary K.L.S., Oldham R.J., Foxall R.B., James S. (2022). HIF activation enhances FcgammaRIIb expression on mononuclear phagocytes impeding tumor targeting antibody immunotherapy. J. Exp. Clin. Cancer Res. CR.

[58] Noy R., Pollard J.W. (2014). Tumor-associated macrophages: From mechanisms to therapy. Immunity.

[59] Ruffell B., Affara N.I., Coussens L.M. (2012). Differential macrophage programming in the tumor microenvironment. Trends Immunol..

[60] Zhang Y., Wang J., Wang W., Tian J., Yin K., Tang X., Ma J., Xu H., Wang S. (2016). IL-17A produced by peritoneal macrophages promote the accumulation and function of granulocytic myeloid-derived suppressor cells in the development of colitis-associated cancer. Tumour Biol. J. Int. Soc. Oncodev. Biol. Med..

[61] Ruffell B., Au A., Rugo H.S., Esserman L.J., Hwang E.S., Coussens L.M. (2012). Leukocyte composition of human breast cancer. Proc. Natl. Acad. Sci. USA.

[62] Henze A.T., Mazzone M. (2016). The impact of hypoxia on tumor-associated macrophages. J. Clin. Investig..

[63] Granata F., Nardicchi V., Loffredo S., Frattini A., Ilaria Staiano R., Agostini C., Triggiani M. (2009). Secreted phospholipases A(2): A proinflammatory connection between macrophages and mast cells in the human lung. Immunobiology.

[64] Ishikawa Y., Komiyama K., Masuda S., Murakami M., Akasaka Y., Ito K., Akishima-Fukasawa Y., Kimura M., Fujimoto A., Kudo I. (2005). Expression of type V secretory phospholipase A in myocardial remodelling after infarction. Histopathology.

[65] Masuda S., Murakami M., Ishikawa Y., Ishii T., Kudo I. (2005). Diverse cellular localizations of secretory phospholipase A2 enzymes in several human tissues. Biochim. Biophys. Acta.

[66] Murakami M., Masuda S., Shimbara S., Ishikawa Y., Ishii T., Kudo I. (2005). Cellular distribution, post-translational modification, and tumorigenic potential of human group III secreted phospholipase A(2). J. Biol. Chem..

[67] Mounier C.M., Wendum D., Greenspan E., Flejou J.F., Rosenberg D.W., Lambeau G. (2008). Distinct expression pattern of the full set of secreted phospholipases A2 in human colorectal adenocarcinomas: sPLA2-III as a biomarker candidate. Br. J. Cancer.

[68] Sved P., Scott K.F., McLeod D., King N.J., Singh J., Tsatralis T., Nikolov B., Boulas J., Nallan L., Gelb M.H. (2004). Oncogenic action of secreted phospholipase A2 in prostate cancer. Cancer Res..

[69] Loffredo S., Borriello F., Iannone R., Ferrara A.L., Galdiero M.R., Gigantino V., Esposito P., Varricchi G., Lambeau G., Cassatella M.A. (2017). Group V Secreted Phospholipase A2 Induces the Release of Proangiogenic and Antiangiogenic Factors by Human Neutrophils. Front. Immunol..

[70] Leung S.Y., Chen X., Chu K.M., Yuen S.T., Mathy J., Ji J., Chan A.S., Li R., Law S., Troyanskaya O.G. (2002). Phospholipase A2 group IIA expression in gastric adenocarcinoma is associated with prolonged survival and less frequent metastasis. Proc. Natl. Acad. Sci. USA.

[71] Tribler L., Jensen L.T., Jorgensen K., Brunner N., Gelb M.H., Nielsen H.J., Jensen S.S. (2007). Increased expression and activity of group IIA and X secretory phospholipase A2 in peritumoral versus central colon carcinoma tissue. Anticancer. Res..

[72] Yamashita S., Yamashita J., Ogawa M. (1994). Overexpression of group II phospholipase A2 in human breast cancer tissues is closely associated with their malignant potency. Br. J. Cancer.

[73] Yamashita S., Yamashita J., Sakamoto K., Inada K., Nakashima Y., Murata K., Saishoji T., Nomura K., Ogawa M. (1993). Increased expression of membrane-associated phospholipase A2 shows malignant potential of human breast cancer cells. Cancer.

[74] Karin M., Cao Y., Greten F.R., Li Z.W. (2002). NF-kappaB in cancer: From innocent bystander to major culprit. Nat. Rev. Cancer.

[75] Mauchley D., Meng X., Johnson T., Fullerton D.A., Weyant M.J. (2010). Modulation of growth in human esophageal adenocarcinoma cells by group IIa secretory phospholipase A(2). J. Thorac. Cardiovasc. Surg..

[76] Ganesan K., Ivanova T., Wu Y., Rajasegaran V., Wu J., Lee M.H., Yu K., Rha S.Y., Chung H.C., Ylstra B. (2008). Inhibition of gastric cancer invasion and metastasis by PLA2G2A, a novel beta-catenin/TCF target gene. Cancer Res..

[77] Xing X.F., Li H., Zhong X.Y., Zhang L.H., Wang X.H., Liu Y.Q., Jia S.Q., Shi T., Niu Z.J., Peng Y. (2011). Phospholipase A2 group IIA expression correlates with prolonged survival in gastric cancer. Histopathology.

[78] Morioka Y., Ikeda M., Saiga A., Fujii N., Ishimoto Y., Arita H., Hanasaki K. (2000). Potential role of group X secretory phospholipase A(2) in cyclooxygenase-2-dependent PGE(2) formation during colon tumorigenesis. FEBS Lett..

[79] Surrel F., Jemel I., Boilard E., Bollinger J.G., Payre C., Mounier C.M., Talvinen K.A., Laine V.J., Nevalainen T.J., Gelb M.H. (2009). Group X phospholipase A2 stimulates the proliferation of colon cancer cells by producing various lipid mediators. Mol. Pharmacol..

[80] Chen Y., Ma X., Lou C., Zhou C., Zhao X., Li N., Tian H., Meng X. (2022). PLA2G10 incorporated in exosomes could be diagnostic and prognostic biomarker for non-small cell lung cancer. Clin. Chim. Acta.

[81] Kudo K., Miki Y., Carreras J., Nakayama S., Nakamoto Y., Ito M., Nagashima E., Yamamoto K., Higuchi H., Morita S.Y. (2022). Secreted phospholipase A2 modifies extracellular vesicles and accelerates B cell lymphoma. Cell Metab..

[82] Tan G., Zhang G.Y., Xu J., Kang C.W., Yan Z.K., Lei M., Pu X.B., Dong C.C. (2020). PLA2G10 facilitates the cell-cycle progression of soft tissue leiomyosarcoma cells at least by elevating cyclin E1/CDK2 expression. Biochem. Biophys. Res. Commun..

[83] Karpisheh V., Nikkhoo A., Hojjat-Farsangi M., Namdar A., Azizi G., Ghalamfarsa G., Sabz G., Yousefi M., Yousefi B., Jadidi-Niaragh F. (2019). Prostaglandin E2 as a potent therapeutic target for treatment of colon cancer. Prostaglandins Other Lipid Mediat..

[84] Chang J., Tang N., Fang Q., Zhu K., Liu L., Xiong X., Zhu Z., Zhang B., Zhang M., Tao J. (2019). Inhibition of COX-2 and 5-LOX regulates the progression of colorectal cancer by promoting PTEN and suppressing PI3K/AKT pathway. Biochem. Biophys. Res. Commun..

[85] Shi D., Feng C., Xie J., Zhang X., Dai H., Yan L. (2022). Recent progress of nanomedicine in secreted phospholipase A2 as a potential therapeutic target. J. Mater. Chem. B.

[86] Triggiani M., Granata F., Frattini A., Marone G. (2006). Activation of human inflammatory cells by secreted phospholipases A2. Biochim. Biophys. Acta.

[87] Granata F., Frattini A., Loffredo S., Del Prete A., Sozzani S., Marone G., Triggiani M. (2006). Signaling events involved in cytokine and chemokine production induced by secretory phospholipase A2 in human lung macrophages. Eur. J. Immunol..

[88] Granata F., Petraroli A., Boilard E., Bezzine S., Bollinger J., Del Vecchio L., Gelb M.H., Lambeau G., Marone G., Triggiani M. (2005). Activation of cytokine production by secreted phospholipase A2 in human lung macrophages expressing the M-type receptor. J. Immunol..

[89] Park D.W., Kim J.R., Kim S.Y., Sonn J.K., Bang O.S., Kang S.S., Kim J.H., Baek S.H. (2003). Akt as a mediator of secretory phospholipase A2 receptor-involved inducible nitric oxide synthase expression. J. Immunol..

[90] Triggiani M., Granata F., Oriente A., De Marino V., Gentile M., Calabrese C., Palumbo C., Marone G. (2000). Secretory phospholipases A2 induce beta-glucuronidase release and IL-6 production from human lung macrophages. J. Immunol..

[91] Ferrara A.L., Galdiero M.R., Fiorelli A., Cristinziano L., Granata F., Marone G., Crescenzo R.M.D., Braile M., Marcella S., Modestino L. (2021). Macrophage-polarizing stimuli differentially modulate the inflammatory profile induced by the secreted phospholipase A2 group IA in human lung macrophages. Cytokine.

[92] Triggiani M., Giannattasio G., Calabrese C., Loffredo S., Granata F., Fiorello A., Santini M., Gelb M.H., Marone G. (2009). Lung mast cells are a source of secreted phospholipases A2. J. Allergy Clin. Immunol..

[93] Murakami M., Masuda S., Shimbara S., Bezzine S., Lazdunski M., Lambeau G., Gelb M.H., Matsukura S., Kokubu F., Adachi M. (2003). Cellular arachidonate-releasing function of novel classes of secretory phospholipase A2s (groups III and XII). J. Biol. Chem..

[94] Rouault M., Le Calvez C., Boilard E., Surrel F., Singer A., Ghomashchi F., Bezzine S., Scarzello S., Bollinger J., Gelb M.H. (2007). Recombinant production and properties of binding of the full set of mouse secreted phospholipases A2 to the mouse M-type receptor. Biochemistry.

